# Hybrid accelerated iterative scheme based on the Picard–Ishikawa approach for systems of nonlinear equations

**DOI:** 10.1371/journal.pone.0355734

**Published:** 2026-09-09

**Authors:** Abubakar Sani Halilu, Hatem E. Semary, Mohamad Afendee Mohamed, Asmaa S. Al-Moisheer, Kabiru Ahmed, Sulaiman M. Ibrahim, Mohammed Yusuf Waziri, Arunava Majumder

**Affiliations:** 1 Faculty of Informatics and Computing, Universiti Sultan Zainal Abidin, Campus Besut, Terengganu, Malaysia; 2 Department of Mathematics, Sule Lamido University Kafin Hausa, Kafin Hausa, Nigeria; 3 Department of Mathematics and Statistics, Faculty of Science, Imam Mohammad Ibn Saud Islamic University (IMSIU), Riyadh, Saudi Arabia; 4 Department of Mathematics, Federal University, Dutse, Nigeria; 5 College of Applied and Health Sciences, A’Sharqiyah University, Ibra, Sultanate of Oman; 6 Department of Mathematical Sciences, Bayero University, Kano, Nigeria; 7 Department of Mathematics, College of Engineering and Technology, SRM Institute of Science and Technology, Kattankulathur, Tamil Nadu, India; Federal University of Technology - Parana, BRAZIL

## Abstract

Systems of nonlinear equations arise in numerous scientific, engineering, and optimization applications, where efficient and reliable numerical methods are required to obtain accurate solutions. Hybridization techniques have recently attracted considerable attention because of their ability to improve the convergence behavior of iterative schemes. Motivated by this development, this paper proposes a novel hybrid accelerated iterative method for solving systems of nonlinear equations by integrating the Picard–Ishikawa process with an accelerated derivative-free framework. The proposed approach employs a diagonal approximation of the Jacobian matrix through an acceleration parameter, thereby reducing computational cost while maintaining numerical accuracy. The novelty of the method lies in combining the three-step Picard–Ishikawa process with an accelerated scheme to enhance convergence performance and robustness. Under standard assumptions, the global convergence of the proposed method is established. Extensive numerical experiments on a collection of benchmark problems demonstrate that the proposed method outperforms several existing approaches in terms of iteration count, computational time, and robustness. These results confirm the effectiveness and suitability of the proposed scheme for solving large-scale systems of nonlinear equations arising in practical applications.

## 1. Introduction

The application of numerical optimization plays an important role in real life. In technology, it is used for making a design and control system [1]. In finance and economics, portfolio selection, risk management, and supply chain logistics rely heavily on these techniques [2]. In health care, its applications include treatment planning in radiation therapy, drug discovery, and medical imaging. It also has applications in energy management, such as optimal power flow in smart grids and the placement of renewable energy systems. Furthermore, in everyday technologies, it is applied in navigation systems, scheduling, and personalized recommendation engines. These real-life applications display the importance of numerical optimization.

Systems of nonlinear equations are one of the basic components of numerical optimization that play a crucial role in a wide range of disciplines, including science and engineering. These equations can model complex phenomena such as fluid dynamics, population, and growth. Given the difficulty in finding exact solutions, researchers often opt for efficient iterative approaches for their approximate solutions that quickly converge accurately, allowing scientists to analyse and predict behaviours in various systems effectively [3–5]. A system of nonlinear equations can be represented as follows:


$$\begin{array}{c} 𝔉(s)=0, \end{array}$$ (1)

where $𝔉:{\mathbb{R}}^{n}\to {\mathbb{R}}^{n}$ is a nonlinear mapping.

The global optimization problem can be derived from (1) [6]. Let 𝔥 be a merit function defined by:


$$\begin{array}{c} 𝔥(s)=\frac{1}{2}\|𝔉(s){\|}^{2}. \end{array}$$ (2)

In this context, the problem of solving the nonlinear Equations (1) is analogous to the following global optimization problem:


$$\begin{array}{c} \min_{s\in {\mathbb{R}}^{n}}𝔥(s),\;𝔥:{\mathbb{R}}^{n}\to \mathbb{R}. \end{array}$$ (3)

Investigators employ several iterative approaches to problem (1), like Newton’s method [7], quasi-Newton methods [8,9], conjugate gradient methods [10,11], accelerated methods [12–14], double direction methods [15–18], and double step length methods [19–22].

Newton’s approach is notable for its appealing characteristics, including simplicity of implementation and rapid convergence. However, the Jacobian matrix can be computed and stored at each iteration. These approaches use the recursive formula to construct a sequence of points:


$$\begin{array}{c} s_{k+1}=s_{k}+\alpha_{k}d_{k},\;k=0,1,..., \end{array}$$ (4)

where $\alpha_{k}$ is the step length, $d_{k}$ is the search direction, and the current and the previous iterations are $s_{k+1}$ and $s_{k}$, respectively. Below is the search direction of Newton’s approach:


$$\begin{array}{c} d_{k}=({𝔉}^{\prime }(s_{k}){)}^{-1}𝔉(s_{k}), \end{array}$$

where ${𝔉}^{\prime }(s_{k})$ is the Jacobian of $𝔉(s_{k})$ at $s_{k}$. One of the main limitations of Newton’s method is its reliance on the Jacobian matrix and its inverse, which must be computed at each iteration. However, the Jacobian may not always be available in closed form or can be expensive and imprecise to evaluate, making the direct application of Newton’s method infeasible in such cases. The calculation of the Jacobian at each iteration makes Newton’s method less applicable for large-scale problems, as they necessitate notable matrix storage, which can be costly in numerical experiments. To address these issues, matrix-free methods have been proposed. For instance, the accelerated gradient method [23] is one of the matrix-free methods for finding the minimizer in (3). The presented method approximated the Hessian matrix via ${▽}^{2}𝔥(s)\approx \delta_{k}I$, where, *I* is an identity matrix, $\delta_{k}>0$ and ${▽}^{2}𝔥(s)$ are the acceleration parameter and the Hessian matrix of $𝔥(s_{k})$, respectively.The rationale of any accelerated method is to develop an effective acceleration parameter. Moreover, in [23], the proposed $\delta_{k}>0$ was derived using the second-order Taylor expansion. Petrovi$\overset{´}{c}$ and Stanimirovi$\overset{´}{c}$ [24] made significant contributions to the field of unconstrained optimisation. Their innovative approach uses an acceleration parameter $\delta_{k}>0$ to create an approximation of the Hessian matrix. The approach in [24] is the extension of the gradient method in [23] by adding the second correction defined as $\alpha_{k}^{2}b_{k}$ in (4) to it. Here $b_{k}$ is the second search direction. The numerical experiments presented in [24] outperformed the one in [23]. In another development, Petrovi$\overset{´}{c}$ [25] introduced a novel double-step length approach by replacing the second correction in [24] with $\beta_{k}b_{k}$, where $\beta_{k}$ is the second step length.

Motivated by the accelerated methods presented for solving (3), several investigators have incorporated this idea to address problem (1) by approximating the Jacobian through ${𝔉}^{\prime }(s_{k})\approx \delta_{k}I$. Employing the first-order Taylor expansion, the acceleration parameter was derived. Building on this idea, Halilu and Waziri [15] invented an accelerated scheme for addressing (1). Thereafter, a modified version of the method [15] was developed in [16]. By assuming that the Jacobian is positive definite and bounded, their study establishes that the proposed method exhibits global convergence. Abdullahi et al. [10] improved the basic methodology from [15] by a conjugate gradient approach for symmetric nonlinear equations. The line search method by Li and Fukushima [6] was employed to achieve global convergence. This innovation represents a significant improvement in the efficiency and applicability for addressing (1). Authors in [18] present another accelerated method for solving (1). The proposed acceleration demonstrates the efficacy of the proposed method in proving global convergence. The extensive numerical results contribute to the ongoing development of robust techniques for solving (1) in an accelerated derivative-free way.

On the other hand, hybrid schemes play a vital role in enhancing the convergence of some iterative methods. As a result, to improve the convergence characteristics and overall numerical efficiency of the accelerated methods, Petrovi$\overset{´}{c}$ et al. [26] studied a hybrid approach that integrates it with the Picard-mann hybrid process presented by Khan in [27]. This idea combines the qualities of both schemes, leading to the performance in solving various unconstrained optimisation problems. The work presented in [26] enhanced the performance of the numerical method of the approach in [24] via the correction parameter. Waziri et al. [12] developed a hybrid method for solving (1) by combining [27] and [19], resulting in the effective acceleration parameter. This advancement demonstrates the benefits of combining multiple schemes to improve overall efficiency in hybrid systems. Following that, Waziri et al. [14] also enhanced the scheme in [15] and produced the correction and accelerated parameters. Motivated by the hybrid method in [12], Liu et al. [28] presented a hybrid acceleration method for solving (1) using hybrid process introduced in [27]. In conclusion, the hybrid approaches demonstrate improved accuracy in obtaining solutions, thereby enhancing the overall effectiveness of the method.

Despite the significant progress achieved by the accelerated and hybrid methods reported in [12,14,22,28], several challenges remain. Most existing approaches are based on the Picard–Mann hybrid process or other two-step schemes, which may not fully exploit the potential advantages of multi-step iterative processes [27]. Furthermore, although these methods have demonstrated satisfactory convergence properties, there is still a need for more efficient derivative-free algorithms with improved robustness and faster convergence for large-scale nonlinear systems. Compared with the existing approaches, the proposed method incorporates the Picard–Ishikawa process [29], a three-step iterative scheme that is known to possess stronger convergence characteristics than the Picard–Mann process. By combining this process with an accelerated Jacobian approximation strategy, the proposed method achieves enhanced numerical performance while maintaining low computational cost. The numerical results presented in this study demonstrate that the proposed scheme consistently performs better than several existing accelerated methods, highlighting its effectiveness and practical relevance.

Many practical problems arising in engineering, applied sciences, and multiphase systems can be formulated as systems of nonlinear equations. Examples include fluid flow models [30–36], heat transfer processes [37], motion control [11,38], and optimisation problems involving coupled physical phenomena [39]. These applications often generate large-scale nonlinear systems for which classical methods may suffer from slow convergence, high computational cost, or difficulties associated with Jacobian evaluations. By reducing computational effort and improving solution accuracy, the proposed method can contribute indirectly to cost reduction, improved resource utilization, and enhanced decision-making in sectors that rely on mathematical modeling and optimization. Although several accelerated and hybrid iterative methods have been proposed in the literature, there remains a need for more efficient derivative-free approaches that combine strong convergence properties with reduced computational complexity. In particular, limited attention has been given to incorporating the Picard–Ishikawa hybrid process into accelerated schemes for solving systems of nonlinear equations. Motivated by this gap, the present study develops a new hybrid accelerated iterative framework that improves convergence performance and numerical efficiency while avoiding direct Jacobian computations. Inspired by concepts introduced in [26,28], we aim to enhance the efficiency and precision of discovering solutions to Problem (1) by hybridising our scheme with the Picard–Ishikawa iterative scheme. These findings not only highlight the potential of the hybrid approach to optimise numerical algorithms but also pave the way for further advancements in this area of study.

The novelty of this work lies in the development of a new Hybrid Accelerated Picard–Ishikawa iterative method for solving systems of nonlinear equations. Unlike existing accelerated methods that primarily employ the Picard–Mann hybrid process or conventional acceleration techniques, the proposed approach integrates the three-step Picard–Ishikawa process with an acceleration framework to improve convergence performance. The main novel contributions of the manuscript are:

- The incorporation of the Picard–Ishikawa hybrid process into an accelerated iterative framework for solving systems of nonlinear equations, which has received limited attention in the existing literature.
- The derivation of a new acceleration parameter that provides an efficient approximation of the Jacobian matrix, leading to a derivative–free implementation and reducing computational cost.
- The introduction of a correction parameter derived from the Picard–Ishikawa process, which enhances the quality of the iterative sequence and improves convergence behavior.
- The establishment of global convergence of the proposed algorithm under mild assumptions.
- Comprehensive numerical experiments demonstrating that the proposed HAPI method consistently outperforms some existing methods, in terms of iteration count, function evaluations, CPU time, and overall robustness.

Therefore, the originality of the study lies not only in proposing a new hybrid accelerated scheme but also in showing that the Picard–Ishikawa-based framework can provide superior numerical performance compared with existing accelerated and hybrid methods for solving large-scale nonlinear systems.

The remainder of this paper is organized as follows. In the next section, the derivation of the proposed approach is presented. A convergence analysis of the suggested algorithm is given in Section 3. The details of the numerical experiments are provided in Section 4. The article is concluded in Section 5. In this paper, all vectors are column vectors and we refer to ${\mathbb{R}}^{n}$ as the *n*-dimensional real space, with the notation ‖·‖ representing the Euclidean norm.

## 2. Motivation and the derivation of the proposed method

In this section, we apply the Picard-Ishikawa hybrid model to an accelerated scheme for solving (1). Let 𝐄 be normed (real) space and let Ω⊂𝐄 be a nonempty convex set. Consider a mapping T:Ω⟶Ω.

The sequence $\left\{u_{k}\right\}$ defines the iterative procedure provided by Picard [40] as


$$\begin{array}{c} \left\{ \begin{array}{c} u_{0}=s\in \Omega ,\\ u_{k+1}=Tu_{k},\;k\geq 0. \end{array}\right. \end{array}$$

The sequence $\left\{v_{k}\right\}$ defines the iterative procedure provided by Mann [41] as


$$\begin{array}{c} \left\{ \begin{array}{c} v_{0}=v\in \Omega ,\\ v_{k+1}=(1-\tau_{k})v_{k}+\tau_{k}Tv_{k},\;k\geq 0, \end{array}\right. \end{array}$$

where $\{\tau_{k}\}\in (0,1).$

The sequences $\left\{y_{k}\right\}$ and $\left\{z_{k}\right\}$ defines the iterative procedure provided by Ishikawa [42] as


$$\begin{array}{c} \left\{ \begin{array}{c} z_{0}=z\in \Omega ,\\ z_{k+1}=(1-\eta_{k})z_{k}+\eta_{k}Ty_{k},\\ y_{k}=(1-\beta_{k})z_{k}+\beta_{k}Tz_{k},\;k\geq 0, \end{array}\right. \end{array}$$

where $\left\{\eta_{k}\right\}$ and $\left\{\beta_{k}\right\}$ are sequences in (0,1).

In [27], Khan hybridised the Picard and Mann iterative methods and presented the Picard-Mann hybrid process with the sequences $\left\{s_{k}\right\}$ and $\left\{z_{k}\right\}$ defined by


$$\begin{array}{c} \left\{ \begin{array}{c} s_{0}=s\in \Omega ,\\ s_{k+1}=Tz_{k},\\ z_{k}=(1-\tau_{k})s_{k}+\tau_{k}Ts_{k},\;k\geq 0, \end{array}\right. \end{array}$$ (5)

where $\left\{\tau_{k}\}\in (0,1\right)$. The presented hybrid process in (12) converges more quickly than all of the Ishikawa, Picard, and Mann, processes. Inspired by the Picard–Mann process, several optimisation schemes have been hybridised with it, yielding improved results compared to the classical methods. For instance, by applying the hybridisation rule described in [27], Petrovi$\overset{´}{c}$ [26] enhanced both the global convergence and the numerical performance of the accelerated method in [24] for solving (3). Similarly, Waziri et al. [12] developed a hybrid technique for solving (1) by incorporating the approaches in [27] and [19]. Numerical experiments displayed that the hybrid method in [12] achieves more rapid convergence than the current method reported in [19]. For further studies in hybridising optimisation techniques with the Picard–Mann process, the reader may refer to [13,14,43,44].

Okeke [29] merged the Picard and Ishikawa procedures to establish the Picard–Ishikawa hybrid approach, which incorporates the simplicity of the Picard process with the ability of the Ishikawa technique. These process is given by


$$\begin{array}{c} \left\{ \begin{array}{c} s_{0}=s\in \Omega ,\\ s_{k+1}=Tz_{k},\\ z_{k}=(1-\eta_{k})s_{k}+\eta_{k}Ty_{k},\\ y_{k}=(1-\beta_{k})s_{k}+\beta_{k}Ts_{k},\;k\geq 0, \end{array}\right. \end{array}$$ (6)

where $\left\{\eta_{k}\right\}$ and $\left\{\beta_{k}\right\}$ are sequences in (0,1). The hybrid process in (6) converges to the solution faster than the Picard, Mann, Ishikawa, and Picard–Mann approaches [29].

Since the Picard–Ishikawa hybrid process exhibits a faster convergence rate than the Picard–Mann hybrid process, we are motivated to develop an accelerated, globally convergent method for solving (1), based on the Picard–Ishikawa hybrid approach.

To begin with, let us define the mapping *T* as follows:


$$\begin{array}{c} Tz_{k}=z_{k}-\alpha_{k}\delta_{k}^{-1}𝔉(s_{k}). \end{array}$$ (7)

By analysing (6) and (7), we can easily deduce the relations that follow:


$$\begin{array}{cc} & s_{0}=s\in \Omega , \end{array}$$

$$\begin{array}{cc} & s_{k+1}=z_{k}-\alpha_{k}\delta_{k}^{-1}𝔉(s_{k}), \end{array}$$ (8)

$$\begin{array}{cc} & z_{k}=(1-\eta_{k})s_{k}+\eta_{k}(y_{k}-\alpha_{k}\delta_{k}^{-1}𝔉(s_{k})) \end{array}$$ (9)

$$\begin{array}{cc} & y_{k}=s_{k}-\alpha_{k}\beta_{k}\delta_{k}^{-1}𝔉(s_{k}),\;k\geq 0. \end{array}$$ (10)

In this study, $\eta_{k}$ and $\beta_{k}$ are regarded as correction parameters that are presented to modify the iteration process and enhance the convergence behaviour of the proposed algorithm.

**Lemma 2.1.** *Suppose the hybrid accelerated approach be generated by the four term iterative process in* (8), (9) *and* (10), *then the proposed iterative scheme is given by*


$$\begin{array}{c} s_{k+1}=s_{k}-\alpha_{k}(\eta_{k}\beta_{k}+\eta_{k}+1)\delta_{k}^{-1}𝔉(s_{k}). \end{array}$$ (11)

**Proof** By substituting (10) into (9) and (9) into (8), we obtain


$$\begin{array}{cc} s_{k+1} & =(1-\eta_{k})s_{k}+\eta_{k}(s_{k}-\alpha_{k}\beta_{k}\delta_{k}^{-1}𝔉(s_{k})-\alpha_{k}\delta_{k}^{-1}𝔉(s_{k}))-\alpha_{k}\delta_{k}^{-1}𝔉(s_{k})\\ & =s_{k}-\alpha_{k}\eta_{k}\beta_{k}\delta_{k}^{-1}𝔉(s_{k})-\alpha_{k}\eta_{k}\delta_{k}^{-1}𝔉(s_{k})-\alpha_{k}\delta_{k}^{-1}𝔉(s_{k})\\ & =s_{k}-\alpha_{k}(\eta_{k}\beta_{k}+\eta_{k}+1)\delta_{k}^{-1}𝔉(s_{k}). \end{array}$$

Therefore, Lemma (2.1) is proved.

### Weighted least-squares update for the acceleration parameter

Given $u_{k-1}=s_{k}-s_{k-1}$ and $v_{k-1}=𝔉(s_{k})-𝔉(s_{k-1})$. If ${𝔉}^{\prime }(s_{k})\approx \delta_{k}I$, then $v_{k-1}\approx {𝔉}^{\prime }(s_{k})u_{k-1}$. Consider the regularised weight matrix


$$\begin{array}{c} W_{k}=v_{k-1}v_{k-1}^{⊤}+\varepsilon I,\;\varepsilon >0. \end{array}$$ (12)

where *I* is an identity matrix.

**Proposition 2.2.** *Let* $v_{k-1}\in {\mathbb{R}}^{n}$*,* $W_{k}\in {\mathbb{R}}^{n\times n}$*, and* ε>0*. Then* $W_{k}$ *has satisfied the following conditions:*

(i) $W_{k}$ *is symmetry*
(ii) $W_{k}$ *is positive definite.*

**Proof (Symmetry)** Being that $(v_{k-1}v_{k-1}^{⊤}{)}^{⊤}=v_{k-1}v_{k-1}^{⊤}$ and $I^{⊤}=I$, it follows that


$$\begin{array}{c} W_{k}^{⊤}=(v_{k-1}v_{k-1}^{⊤}{)}^{⊤}+(\varepsilon I{)}^{⊤}=v_{k-1}v_{k-1}^{⊤}+\varepsilon I=W_{k}. \end{array}$$

Hence the result follows.

**(Positive definiteness).** For any nonzero vector $x\in {\mathbb{R}}^{n}$,


$$\begin{array}{c} x^{⊤}W_{k}x=x^{⊤}(v_{k-1}v_{k-1}^{⊤})x+\varepsilon x^{⊤}x=(v_{k-1}^{⊤}x{)}^{2}+\varepsilon \|x{\|}^{2}. \end{array}$$

Clearly $(v_{k-1}^{⊤}x{)}^{2}\geq 0$, and since ε>0 and $\|x{\|}^{2}>0$ for x≠0, we have


$$\begin{array}{c} x^{⊤}W_{k}x>0. \end{array}$$

Hence the result follows.

To obtain the acceleration parameter $\delta_{k}>0$, the following weighted least squares problem can be formulated:


$$\begin{array}{c} \min_{\delta \in \mathbb{R}}\;\|\;v_{k-1}-\delta u_{k-1}\;{\|}_{W_{k}}^{2}=\min_{\delta }(v_{k-1}-\delta u_{k-1}{)}^{⊤}W_{k}(v_{k-1}-\delta u_{k-1}). \end{array}$$

Define


$$\begin{array}{c} \varphi (\delta )=(v_{k-1}-\delta u_{k-1}{)}^{⊤}W_{k}(v_{k-1}-\delta u_{k-1}). \end{array}$$ (13)

Differentiating (13) with respect to δ and setting the result to zero yields


$$\begin{array}{c} \varphi^{\prime }(\delta )=-2u_{k-1}^{⊤}W_{k}v_{k-1}+2\delta u_{k-1}^{⊤}W_{k}u_{k-1}=0. \end{array}$$ (14)

By substituting (12) into (14), we obtain


$$\begin{array}{c} \delta [u_{k-1}^{⊤}(v_{k-1}v_{k-1}^{⊤}+\varepsilon I)u_{k-1})]=u_{k-1}^{⊤}(v_{k-1}v_{k-1}^{⊤}+\varepsilon I)v_{k-1}). \end{array}$$

After a little algebraic simplification,


$$\begin{array}{c} \delta [(v_{k-1}^{⊤}u_{k-1}{)}^{2}+\varepsilon \|u_{k-1}{\|}^{2}]=(v_{k-1}^{⊤}u_{k-1})\|v_{k-1}{\|}^{2}+\varepsilon (v_{k-1}^{⊤}u_{k-1}). \end{array}$$

Therefore, the acceleration parameter yields


$$\begin{array}{c} \delta_{k}=\frac{v_{k-1}^{⊤}u_{k-1}(\|v_{k-1}{\|}^{2}+\varepsilon )}{(v_{k-1}^{⊤}u_{k-1}{)}^{2}+\varepsilon \|u_{k-1}{\|}^{2}}. \end{array}$$ (15)

From (11), the proposed search direction is expressed as:


$$\begin{array}{c} d_{k}=-(\eta_{k}\beta_{k}+\eta_{k}+1)\delta_{k}^{-1}𝔉(s_{k}). \end{array}$$ (16)

The general scheme can be stated as follows:


$$\begin{array}{c} s_{k+1}=s_{k}+\alpha_{k}d_{k}. \end{array}$$

**Remark 2.3.** *We make the following observations:*

(i) *The sign of the proposed acceleration parameter in (15) corresponds the sign of* $v_{k-1}^{⊤}u_{k-1}$*. Specifically* $\delta_{k}>0$ *whenever* $v_{k-1}^{⊤}u_{k-1}>0$.
(ii) *Since* $W_{k}≻0$*, the denominator in (15) is strictly positive when* $u_{k-1}\text{≠}0$*; therefore, the minimiser is unique. In addition,* $\varphi^{\prime \prime }(\delta )=2\;u_{k-1}^{⊤}W_{k}u_{k-1}>0$*, hence* φ *is strictly convex in* δ.
(iii) *We specify the correction parameters* $\eta_{k}=\eta \in (0,1)$ *and* $\beta_{k}=\beta \in (0,1)$ *for all k > 0, thereby assuring consistency in the analysis and allowing effective modification of the proposed iterative process.*

**Algorithm 1: Hybrid Accelerated Scheme Based on the Picard–Ishikawa (HAPI)**

**Input:** Given *s*_0_, $\delta_{0}=1$, $\epsilon =10^{-5}$, $\omega_{1},\;\omega_{2}>0,$ ρ,η,β,θ∈(0,1), and set *k*=0.

**1:** Compute $𝔉(s_{k}).$

**2:** If $\|𝔉(s_{k})\|\leq \epsilon$ then stop; otherwise, continue to **3**.

**3:** Determine the search direction $d_{k}=-(\eta \beta +\eta +1)\delta_{k}^{-1}𝔉(s_{k})$.

**4:** Establish $s_{k+1}=s_{k}+\alpha_{k}d_{k}$, with, $\alpha_{k}=\rho^{m_{k}}$ and $m_{k}$ denotes the smallest nonnegative integer *m* such that

$𝔥(s_{k}+\alpha_{k}d_{k})-𝔥(s_{k})\leq -\omega_{1}\|\alpha_{k}𝔉(s_{k}){\|}^{2}-\omega_{2}\|\alpha_{k}d_{k}{\|}^{2}+\chi_{k}𝔥(s_{k}).$ (17)

Suppose that $\left\{\chi_{k}\right\}$ is a positive sequence such that

$\sum_{k=0}^{\infty }\chi_{k}<\chi <\infty .$ (18)

**5:** Compute $𝔉(s_{k+1})$.

**6:** Determine $\delta_{k+1}=\frac{v_{k}^{⊤}u_{k}(\|v_{k}{\|}^{2}+\varepsilon )}{(v_{k}^{⊤}u_{k}{)}^{2}+\varepsilon \|u_{k}{\|}^{2}}$.

**7:** Consider *k*=*k*+1 and go to **2**.

**Lemma 2.4.** *Suppose that the hybrid accelerated approach is generated by the Picard–Mann hybrid process in (5). Then, the iterative scheme can be expressed as*


$$\begin{array}{c} s_{k+1}=s_{k}-\alpha_{k}(\theta_{k}+1)\delta_{k}^{-1}𝔉(s_{k}). \end{array}$$ (19)

**Proof** The proof follows the same argument as that of Lemma 2.1. Hence, we obtain (19).

**Remark 2.5.** *From (19), it follows that the search direction of the hybrid accelerated scheme based on the Picard–Mann process (HAPM) is given by*


$$\begin{array}{c} d_{k}^{HAPM}=-(\theta_{k}+1)\delta_{k}^{-1}𝔉(s_{k}). \end{array}$$

Following a similar approach to Remark 2.3, we choose the correction parameter as a constant, that is, $\theta_{k}=\theta \in (0,1)$ for all k≥0. Therefore, by replacing the search direction in Step 3 of Algorithm 1 (HAPI) with $\left(d_{k}^{HAPM}\right)$, we obtain Algorithm 2 (HAPM).

## 3. Convergence analysis

The global convergence of the proposed Algorithm 1 (HAPI) is established in this section. We begin by defining the level set.


$$\begin{array}{c} \Omega =\{s|\|𝔉(s)\|\leq \|𝔉(s_{0})\|\}. \end{array}$$ (20)

**Assumption 3.1.** *The following assumptions have been stated:*

1. *There exists* $s^{*}\in {\mathbb{R}}^{n}$ *such that* $𝔉(s^{*})=0$.
2. *The function* 𝔉(s) *is continuously differentiable in some neighborhood say Q of* $s^{*}$ *containing* Ω.
3. *The Jacobian of* 𝔉(s) *is bounded on Q, i.e., there exist a constant H > 0 such that*
  $$\begin{array}{c} \|{𝔉}^{\prime }(s)\|\leq H,\;\forall s\in Q. \end{array}$$ (21)
4. *The Jacobian of* 𝔉(s) *is positive definite on Q, i.e., there exist a constant h > 0 such that*
  $$\begin{array}{c} h\|d{\|}^{2}\leq d^{T}{𝔉}^{\prime }(s)d,\;\forall s\in Q,d\in {\mathbb{R}}^{n}. \end{array}$$ (22)

*Consequently, we have*


$$\begin{array}{c} h\|d\|\leq \|{𝔉}^{\prime }(s)d\|,\;\forall s\in Q,d\in {\mathbb{R}}^{n}. \end{array}$$

**Remark 3.2.** *We make the following remark:*

*Assumption 3.1 implies that*


$$\begin{array}{c} h\|d\|\leq \|{𝔉}^{\prime }(s)d\|\leq H\|d\|,\;\forall s\in Q,d\in {\mathbb{R}}^{n}. \end{array}$$ (23)

$$\begin{array}{c} h\|s-v\|\leq \|𝔉(s)-𝔉(v)\|\leq H\|s-v\|\;\forall s,v\in Q. \end{array}$$ (24)

**Assumption 3.3.** $(\eta \beta +\eta +1{)}^{-1}\delta_{k}I$ *is a good approximation to* ${𝔉}^{\prime }(s_{k})$*, i.e.,*


$$\begin{array}{c} \|({𝔉}^{\prime }(s_{k})-(\eta \beta +\eta +1{)}^{-1}\delta_{k}I)d_{k}\|\leq \mu \|𝔉(s_{k})\|, \end{array}$$ (25)

*where,* μ∈(0,1) *is a small quantity.*

**Lemma 3.4.** *Let* $\left\{s_{k}\right\}$ *be produced by the HAPI algorithm, supposing Assumption 3.3 is true. Then* $d_{k}$ *is a sufficient descent direction for* $𝔥(s_{k})$ *at* $s_{k}$*, i.e.,*


$$\begin{array}{c} ▿𝔥(s_{k}{)}^{T}d_{k}\leq -c\|𝔉(s_{k}){\|}^{2},\;c>0. \end{array}$$ (26)

**Proof** From (2), (16), and (25), we have


$$\begin{array}{cc} ▿𝔥(s_{k}{)}^{T}d_{k} & ={𝔉}_{k}^{T}{𝔉}^{\prime }(s_{k})d_{k}\\ & =𝔉(s_{k}{)}^{T}[({𝔉}^{\prime }(s_{k})-(\eta \beta +\eta +1{)}^{-1}\delta_{k}I)d_{k}-𝔉(s_{k})]\\ & =𝔉(s_{k}{)}^{T}({𝔉}^{\prime }(s_{k})-(\eta \beta +\eta +1{)}^{-1}\delta_{k}I)d_{k}-\|𝔉(s_{k}){\|}^{2}. \end{array}$$

Applying the Cauchy-Schwarz inequality, we have,


$$\begin{array}{cc} ▿𝔥(s_{k}{)}^{T}d_{k} & \leq \|𝔉(s_{k})\|\|({𝔉}^{\prime }(s_{k})-(\eta \beta +\eta +1{)}^{-1}\delta_{k}I)d_{k}\|-\|𝔉(s_{k}){\|}^{2}\\ & \leq -(1-\mu )\|𝔉(s_{k}){\|}^{2}. \end{array}$$

Since μ∈(0,1), taking c=1−μ, Lemma 3.4 is true.

From Lemma 3.4, we conclude that, $\left\|𝔉(s_{k+1})\|\leq \|𝔉(s_{k})\right\|$ is true for all *k*.

**Lemma 3.5.** *Let* $\left\{s_{k}\right\}$ *be produced by the HAPI algorithm, supposing Assumption 3.3 is true. Then* $\{s_{k}\}\subset \Omega$*.*

**Proof** From Lemma 3.4, we have $\left\|𝔉(s_{k+1})\|\leq \|𝔉(s_{k})\right\|$. Furthermore, for all *k*,


$$\begin{array}{c} \|𝔉(s_{k+1})\|\leq \|𝔉(s_{k})\|\leq \|𝔉(s_{k-1})\|\leq ...\leq \|𝔉(s_{0})\|. \end{array}$$

This means that $\{s_{k}\}\subset \Omega$.

**Lemma 3.6.** *Let* $\left\{s_{k}\right\}$ *be produced by the HAPI algorithm, supposing Assumption 3.3 is true. Then there exists a constant h > 0 such that for all k,*


$$\begin{array}{c} v_{k}^{T}u_{k}\geq h\|u_{k}{\|}^{2}. \end{array}$$ (27)

**Proof** From mean-value theorem and (22),


$$\begin{array}{c} v_{k}^{T}u_{k}=u_{k}^{T}(𝔉(s_{k+1})-𝔉(s_{k}))=u_{k}^{T}F^{\prime }(\zeta )u_{k}\geq h\|u_{k}{\|}^{2}. \end{array}$$

Where $\zeta =s_{k}+\psi (s_{k+1}-s_{k})$, ψ∈(0,1).

**Proposition 3.7.** *From (15), (24), and (27), we obtain the following cases:*


$$\begin{array}{c} \delta_{k}=\frac{v_{k-1}^{⊤}u_{k-1}(\|v_{k-1}{\|}^{2}+\varepsilon )}{(v_{k-1}^{⊤}u_{k-1}{)}^{2}+\varepsilon \|u_{k-1}{\|}^{2}}\geq \frac{h\|u_{k-1}{\|}^{2}(\|v_{k-1}{\|}^{2}+\varepsilon )}{\|u_{k-1}{\|}^{2}\|v_{k-1}{\|}^{2}+\varepsilon \|u_{k-1}{\|}^{2}}=\frac{h\|u_{k-1}{\|}^{2}(\|v_{k-1}{\|}^{2}+\varepsilon )}{\left\|u_{k-1}{\|}^{2}(\|v_{k-1}{\|}^{2}+\varepsilon \right)}=h. \end{array}$$

*This implies that*


$$\begin{array}{c} |\delta_{k}|\geq h. \end{array}$$ (28)

*Consequently,*


$$\begin{array}{c} |\delta_{k}^{-1}|\leq \frac{1}{h}. \end{array}$$ (29)

**Lemma 3.8.** *Let* $\left\{s_{k}\right\}$ *be produced by the HAPI algorithm, supposing Assumption 3.3 is true. Then for all k, we have*


$$\begin{array}{c} \lim_{k\to \infty }\|\alpha_{k}d_{k}\|=\lim_{k\to \infty }\|u_{k}\|=0, \end{array}$$ (30)

*and*


$$\begin{array}{c} \lim_{k\to \infty }\|\alpha_{k}𝔉(s_{k})\|=0. \end{array}$$ (31)

**Proof** From (17) for all *k* > 0


$$\begin{array}{cc} \omega_{2}\|\alpha_{k}d_{k}{\|}^{2} & \leq \omega_{1}\|\alpha_{k}𝔉(s_{k}){\|}^{2}+\omega_{2}\|\alpha_{k}d_{k}{\|}^{2}\\ & \leq \|𝔉(s_{k}){\|}^{2}-\|𝔉(s_{k+1}){\|}^{2}+\chi_{k}\|𝔉(s_{k}){\|}^{2}. \end{array}$$

By summing the above inequality, we have


$$\begin{array}{cc} \omega_{2}\sum_{i=0}^{k}\|\alpha_{i}d_{i}{\|}^{2} & \leq \sum_{i=0}^{k}\left(\|𝔉(s_{i}){\|}^{2}-\|𝔉(s_{i+1}){\|}^{2}\right)+\sum_{i=0}^{k}\chi_{i}\|𝔉(s_{i}){\|}^{2},\\ & =\|𝔉(s_{0)}{\|}^{2}-\|𝔉(s_{k+1}){\|}^{2}+\sum_{i=0}^{k}\chi_{i}\|𝔉(s_{i}){\|}^{2},\\ & \leq \|𝔉(s_{0}){\|}^{2}+\|𝔉(s_{0}){\|}^{2}\sum_{i=0}^{k}\chi_{i},\\ & \leq \|𝔉(s_{0}){\|}^{2}+\|𝔉(s_{0}){\|}^{2}\sum_{i=0}^{\infty }\chi_{i}. \end{array}$$

Based on the level set and the fact that the sequence $\left\{\chi_{k}\right\}$ meets the criterion in (18), it follows that the series $\sum_{i=0}^{\infty }\|\alpha_{i}d_{i}{\|}^{2}$ converges. This leads to the conclusion in (30). By applying the same reasoning as above, but now considering $\omega_{1}\|\alpha_{k}{𝔉}_{k}{\|}^{2}$ on the left side, we arrive at (31).

**Lemma 3.9.** *Let* $\left\{s_{k}\right\}$ *be produced by the HAPI algorithm, supposing Assumption 3.3 is true. Then there exists a constant M_1_ > 0 such that for all k > 0,*


$$\begin{array}{c} \|d_{k}\|\leq M_{1}. \end{array}$$ (32)

**Proof** From Remark 2.3 (iii), the level set, (16), (24), and (29), we have


$$\begin{array}{cc} \left\|d_{k}\right\| & =\|-(\eta \beta +\eta +1)\delta_{k}^{-1}𝔉(s_{k})\|\\ & \leq (\eta \beta +\eta +1)|\delta_{k}^{-1}|\|𝔉(s_{k})\|\\ & \leq (\eta \beta +\eta +1)\frac{1}{h}\|𝔉(s_{k})\|\\ & \leq (\eta \beta +\eta +1)\frac{1}{h}\|𝔉(s_{0})\|. \end{array}$$

Taking $M_{1}=(\eta \beta +\eta +1)\frac{1}{h}\|𝔉(s_{0})\|$, we have (32).

**Lemma 3.10.** *Let* $\left\{s_{k}\right\}$ *be produced by the HAPI algorithm, supposing Assumption 3.3 is true. Then there exists a constant B_1_ > 0 such that for all k > 0,*


$$\begin{array}{c} |\delta_{k}|\leq B_{1}. \end{array}$$ (33)

**Proof** From (24), we obtain


$$\begin{array}{c} h\|u_{k-1}{\|}^{2}\leq \|v_{k-1}\|\|u_{k-1}\|\leq H\|u_{k-1}{\|}^{2}. \end{array}$$ (34)

Combining (27) and (34) gives


$$\begin{array}{c} h\|u_{k-1}{\|}^{2}\leq v_{k-1}^{⊤}u_{k-1}\leq H\|u_{k-1}{\|}^{2}. \end{array}$$ (35)

Now, From the the definition of $\delta_{k}$ in (15) and applying (35), it follows that


$$\begin{array}{c} |\delta_{k}|=\left|\frac{v_{k-1}^{⊤}u_{k-1}(\|v_{k-1}{\|}^{2}+\varepsilon )}{(v_{k-1}^{⊤}u_{k-1}{)}^{2}+\varepsilon \|u_{k-1}{\|}^{2}}\right|\leq \frac{H\|u_{k-1}{\|}^{2}(H^{2}\|u_{k-1}{\|}^{2}+\varepsilon )}{\varepsilon \|u_{k-1}{\|}^{2}}\leq \frac{H(H^{2}\|u_{k-1}{\|}^{2}+\varepsilon )}{\varepsilon }. \end{array}$$

From Lemma 3.8, we know that $\lim_{k\to \infty }\|u_{k-1}\|=0$. Hence, there exists a positive constant *m*_3_ such that $\|u_{k-1}\|\leq m_{3}$ for all *k*.

Therefore,


$$\begin{array}{c} |\delta_{k}|\leq \frac{H(H^{2}m_{3}^{2}+\varepsilon )}{\varepsilon }. \end{array}$$

Setting


$$\begin{array}{c} B_{1}=\frac{H(H^{2}m_{3}^{2}+\varepsilon )}{\varepsilon }, \end{array}$$

we obtain (33).

**Corollary 3.11.** *From Lemma 3.10, and fact that* $\delta_{k}>0$ *for all k, we have*


$$\begin{array}{c} |\delta_{k}^{-1}|\geq \frac{1}{B_{1}}\;\text{for all }k. \end{array}$$ (36)

*In addition, from (28) there exists h > 0 with* $|\delta_{k}|\geq h$ *for all k, then*


$$\begin{array}{c} \frac{1}{B_{1}}\leq |\delta_{k}^{-1}|\leq \frac{1}{h}. \end{array}$$

**Theorem 3.12.** *Let* $\left\{s_{k}\right\}$ *be produced by the HAPI algorithm, supposing Assumption 3.3 is true, then*


$$\begin{array}{c} \lim_{k\to \infty }\|𝔉(s_{k})\|=0. \end{array}$$ (37)

**Proof** From (30), either


$$\begin{array}{c} \lim_{k\to \infty }\|d_{k}\|=0 \end{array}$$ (38)

or


$$\begin{array}{c} \lim_{k\to \infty }\alpha_{k}=0, \end{array}$$

If (38) holds, then from (16) we have,


$$\begin{array}{c} 𝔉(s_{k}{)}^{T}d_{k}=-(\eta \beta +\eta +1)\delta_{k}^{-1}\|𝔉(s_{k}){\|}^{2}. \end{array}$$ (39)

From (39), and Cauchy-Schwarz inequality, we obtain


$$\begin{array}{c} \|d_{k}\|\geq (\eta \beta +\eta +1)|\delta_{k}^{-1}|\|𝔉(s_{k})\|. \end{array}$$ (40)

Also, (40) and (36) gives


$$\begin{array}{c} \|d_{k}\|\geq (\eta \beta +\eta +1)\frac{1}{B_{1}}\|𝔉(s_{k})\|. \end{array}$$

As a result,


$$\begin{array}{c} 0\leq (\eta \beta +\eta +1)\frac{1}{B_{1}}\|𝔉(s_{k})\|\leq \|d_{k}\|⟶0. \end{array}$$

Hence, (37) holds.

On the other hand, if (38) does not hold, then there exists a constant ϖ>0 such that


$$\begin{array}{c} \|d_{k}\|\geq \varpi ,\;\forall k\geq 0. \end{array}$$ (41)

Since 𝔉(s) is continuously differentiable and $𝔥(s)=\frac{1}{2}\|𝔉(s){\|}^{2}$, we can use the first-order Taylor expansion to derive results as follows:


$$\begin{array}{c} 𝔥(s_{k}+\rho^{-1}\alpha_{k}d_{k})-𝔥(s_{k})=\rho^{-1}\alpha_{k}▿𝔥(s_{k}{)}^{T}d_{k}+o(\rho^{-1}\alpha_{k}\|d_{k}\|). \end{array}$$ (42)

From (17),


$$\begin{array}{c} 𝔥(s_{k}+\rho^{-1}\alpha_{k}d_{k})-𝔥(s_{k})>-\omega_{1}\|\rho^{-1}\alpha_{k}𝔉(s_{k}){\|}^{2}-\omega_{2}\|\rho^{-1}\alpha_{k}d_{k}{\|}^{2}+\chi_{k}𝔥(s_{k}). \end{array}$$ (43)

From (42) and (43), and using (26), the following inequality can be established.


$$\begin{array}{c} \omega_{1}\|\alpha_{k}𝔉(s_{k}){\|}^{2}+\omega_{2}\|\alpha_{k}d_{k}{\|}^{2}\geq \rho c\alpha_{k}\|𝔉(s_{k}){\|}^{2}-o(\rho \alpha_{k}\|d_{k}\|). \end{array}$$ (44)

Dividing (44) through by $\alpha_{k}\|d_{k}\|$, we can easily derive the following results:


$$\begin{array}{c} \omega_{1}\alpha_{k}\frac{\|𝔉(s_{k}){\|}^{2}}{\left\|d_{k}\right\|}+\omega_{2}\alpha_{k}\|d_{k}\|\geq \rho c\frac{\|𝔉(s_{k}){\|}^{2}}{\left\|d_{k}\right\|}-\rho \frac{o(\alpha_{k}\|d_{k}\|)}{\alpha_{k}\|d_{k}\|}. \end{array}$$

Therefore, from (38), (41), one can readily derive the following conclusion.


$$\begin{array}{c} \lim_{k\to \infty }\frac{\|𝔉(s_{k}){\|}^{2}}{\left\|d_{k}\right\|}=0. \end{array}$$

Therefore, we have (37). The proof is complete.

**Remark 4.** *By employing the search direction* $d_{k}^{HAPM}$ *defined in (5), the convergence analysis of the HAPM algorithm can be established in a manner analogous to that of the HAPI algorithm. Consequently, the corresponding convergence results hold for the HAPM algorithm.*

## 4. Numerical experiments

This section provides an overview of the results acquired from numerical experiments on a system of nonlinear equations. The objective of these experiments is to evaluate the performance and effectiveness of the proposed method in solving (1). The efficiency of the proposed approach is compared with two recent algorithms from [28], specifically ALG1 and ALG2.

The computer codes employed in this study were developed using MATLAB version 9.4.0 (R2020a) and executed on a personal computer that features a 1.80 GHz CPU processor and 8 GB of RAM. All four algorithms employed the same line search method, as detailed in (17), to ensure a fair and consistent performance comparison. Throughout the numerical experiments, the parameters for ALG1 and ALG2 were adopted from the settings reported in [28]. The parameter settings used for the HAPI and HAPM algorithms are reported in Table 1. The program execution terminates if the total number of iterations exceeds 1000 or if $\|𝔉(s_{k})\|\leq 10^{-5}$. The symbol ’–’ denotes a failure when the number of iterations exceeds 1000 without discovering a point of $s_{k}$ that meets the established stopping criterion. To provide the comprehensive numerical experiments conducted with the three methods, we applied these techniques to eight benchmark test problems, using various initial points and dimensions ranging from 1000 to 100,000. The initial points used in the experiment are: $s_{0}^{1}={\left(\frac{1}{2},\frac{1}{2},...,\frac{1}{2}\right)}^{T}$, $s_{0}^{2}={\left(\frac{1}{5},\frac{1}{5},...,\frac{1}{5}\right)}^{T}$, $s_{0}^{3}={\left(\frac{3}{2},\frac{3}{2},...,\frac{3}{2}\right)}^{T}$, $s_{0}^{4}={\left(\frac{2}{5},\frac{2}{5},...,\frac{2}{5}\right)}^{T}$, $s_{0}^{5}={\left(0,\frac{1}{2},\frac{2}{3},...,1-\frac{1}{n}\right)}^{T}$, and $s_{0}^{6}={\left(1,\frac{1}{2},\frac{1}{3},...,\frac{\left(1\right)}{n}\right)}^{T}$.

**Table 1:** Summary of benchmark problems and experimental settings.

Problem	Description	Parameter(s)	Reference
P1	Polynomial nonlinear system with coupled variables	–	[28]
P2	Trigonometric nonlinear system	–	[15]
P3	Cubic nonlinear system with nearest-neighbour coupling	–	[28]
P4	Integral-type nonlinear system	*c* = 0.1	[21]
P5	Exponential–trigonometric nonlinear system	–	[17]
P6	Exponential-cosine nonlinear system	–	[12]
P7	Quadratic nonlinear system	–	[12]
P8	Exponential nonlinear system	–	[19]
**Common algorithmic parameters: $\omega_{1}=\omega_{2}=10^{-4},\;\rho =0.2,\;\varepsilon =0.01,\;\chi_{k}=\frac{1}{(k+1{)}^{2}}$**			
HAPI: β=0.01, η=0.001			
HAPM: θ=0.1			
Dimensions tested: n∈{1,000,10,000,100,000}			

The benchmark problems and the corresponding experimental settings used to evaluate the performance of the algorithms are summarized in Table 1. Detailed definitions of the test problems are provided below.

**Problem 1** [28]

${𝔉}_{i}(s)=(1-s_{i}^{2})+s_{i}(1+s_{i}s_{n-2}s_{n-1}s_{n})-2$, *i*=1,2,...,*n*.

**Problem 2** [15]

${𝔉}_{i}(s)=s_{i}-3s_{i}\left(\frac{\sin s_{i}}{3}-\frac{33}{50}\right)+2$, *i*=1,2,...,*n*.

**Problem 3** [28]

${𝔉}_{1}(s)=s_{1}(s_{1}^{2}+s_{2}^{2})-1,$

${𝔉}_{i}(s)=s_{i}(s_{i-1}^{2}+2s_{i}^{2}+s_{i+1}^{2})-1,$

${𝔉}_{n}(s)=s_{n}(s_{n-1}^{2}+s_{n}^{2})$, i=2,3,⋯,n−1.

**Problem 4** [21]

${𝔉}_{i}(s)=s_{i}-{(1-\frac{c}{2n}\sum_{j=1}^{n}\frac{\mu_{i}s_{j}}{\mu_{i}+\mu_{j}})}^{-1}$, i=1,2,...,n,j=1,2,...,n.

with c∈[0,1) and $\mu =\frac{i-0.5}{n}$. (In our experiment, we take c = 0.1).

**Problem 5** [17]

${𝔉}_{1}(s)=s_{1}-e^{\cos\left(\frac{s_{1}+s_{2}}{n+1}\right)}$,

${𝔉}_{i}(s)=s_{i}-e^{\cos\left(\frac{s_{i-1}+s_{i}+s_{i+1}}{n+1}\right)}$,

${𝔉}_{n}(s)=s_{n}-e^{\cos\left(\frac{s_{n-1}+s_{n}}{n+1}\right)}$, i=2,3,...,n−1.

**Problem 6** [12]

${𝔉}_{i}(s)=e^{s_{i}^{2}-1}-\cos(1-s_{i})$ i=1,2,⋯,n.

**Problem 7** [12]

${𝔉}_{i}(s)=s_{i}^{2}-1$, i=1,2,⋯,n,

**Problem 8** [19]

${𝔉}_{1}(s)=\cos s_{1}-9+3s_{1}+8e^{s_{1}}$,

${𝔉}_{i}(s)=\cos s_{i}-9+3s_{i}+8e^{s_{i}}$, i=2,3,⋯,n.

Table 1 provides a summary of the benchmark problems and the parameter settings employed throughout the numerical experiments.

The numerical comparisons reported in Tables 2–6 were conducted using the benchmark problems and parameter settings summarized in Table 1. Tables 2–5 display the performance of the HAP1 method against the ALG1 and ALG2 methods for problems 1–8. The evaluation criteria include the number of iterations, function evaluations, CPU time, and the residual norm $\left\|𝔉(s_{k})\right\|$. For all problems, the HAPI method consistently achieves convergence with fewer iterations and function evaluations than the ALG1 and ALG2 methods. As the problem size increases, the advantages of the HAPI methods become more apparent, particularly in cases where the ALG1 and ALG2 methods have failed, as illustrated in Table 3. Additionally, the CPU time supports this observation: HAPI generally requires less computational effort, whereas the competing methods demonstrate significantly longer execution times, especially in large-scale scenarios. All three methods achieve similar residual norms that stay within acceptable tolerances regarding solution accuracy. However, the HAPI approach displays better accuracy and efficiency than the compared methods. Therefore, approximating the Jacobian along with the acceleration parameter significantly reduces the computational burden of the proposed method. Overall, the results in Tables 2–5 demonstrate that the hybrid accelerated method is not only faster but also more robust for large-scale nonlinear systems. Its outstanding performance using three metrics, i.e., number of iterations, function evaluations, and CPU time, validates its usefulness compared to existing approaches.

**Table 2:** Numerical experiments for problems 1 & 2 with respect to HAPI, ALG1 & ALG2 methods.

Problem	Dim	IP	HAPI				ALG1				ALG2			
			Iter	Fun	Time(s)	$\left\|𝔉(s_{k})\right\|$	Iter	Fun	Time(s)	$\left\|𝔉(s_{k})\right\|$	Iter	Fun	Time(s)	$\left\|𝔉(s_{k})\right\|$
P1	1,000	s1	7	11	0.01651	1.82E-08	12	17	0.01402	5.16E-06	12	17	0.01810	5.16E-06
		s2	5	7	0.01083	3.21E-06	12	17	0.01382	2.42E-06	12	17	0.01126	2.42E-06
		s3	8	12	0.00741	3.50E-06	14	19	0.00552	5.31E-06	14	19	0.00631	5.31E-06
		s4	9	11	0.00559	2.86E-08	12	17	0.00766	2.12E-06	12	17	0.00706	2.12E-06
		s5	7	10	0.01265	8.28E-06	17	21	0.00929	4.51E-06	17	23	0.00925	6.98E-06
		s6	8	11	0.00650	5.21E-06	21	30	0.00796	9.86E-06	19	28	0.00845	7.95E-06
	10,000	s1	7	11	0.02170	5.74E-08	13	18	0.03124	3.26E-06	13	18	0.03348	3.26E-06
		s2	6	8	0.01476	9.75E-09	12	17	0.03305	7.64E-06	12	17	0.03366	7.64E-06
		s3	9	13	0.02713	1.14E-08	15	20	0.03527	3.36E-06	15	20	0.03705	3.36E-06
		s4	9	11	0.02254	9.04E-08	12	17	0.03323	6.69E-06	12	17	0.03204	6.69E-06
		s5	5	7	0.01397	1.04E-06	19	22	0.04348	9.65E-06	19	23	0.04615	9.78E-06
		s6	13	18	0.03406	8.87E-06	20	27	0.04851	7.49E-06	23	33	0.05500	9.41E-06
	100,000	s1	7	11	0.11364	1.82E-07	14	19	0.19404	2.06E-06	14	19	0.19568	2.06E-06
		s2	6	8	0.08753	3.08E-08	13	18	0.19428	4.83E-06	13	18	0.18385	4.83E-06
		s3	9	13	0.15968	3.62E-08	16	21	0.23544	2.12E-06	16	21	0.21831	2.12E-06
		s4	9	11	0.12301	2.86E-07	13	18	0.19320	4.23E-06	13	18	0.18704	4.23E-06
		s5	5	7	0.07666	9.93E-07	16	22	0.23806	8.81E-06	17	23	0.22937	7.25E-06
		s6	9	12	0.12349	1.76E-06	23	32	0.32885	6.98E-06	21	31	0.29615	9.38E-06
P2	1,000	s1	6	8	0.00534	1.50E-07	11	13	0.00674	8.36E-06	11	13	0.00850	8.36E-06
		s2	5	7	0.00346	5.22E-06	11	13	0.00644	6.46E-06	11	13	0.00557	6.46E-06
		s3	7	10	0.00417	2.81E-06	10	12	0.00746	6.54E-06	10	12	0.00638	6.54E-06
		s4	6	8	0.00402	5.15E-08	11	13	0.00751	7.82E-06	11	13	0.00724	7.82E-06
		s5	7	9	0.00604	1.31E-08	11	13	0.00824	7.95E-06	11	13	0.00745	7.96E-06
		s6	5	7	0.00361	4.82E-07	11	13	0.00806	4.96E-06	11	13	0.00693	4.96E-06
	10,000	s1	6	8	0.01664	4.73E-07	12	14	0.03180	5.29E-06	12	14	0.03134	5.29E-06
		s2	6	8	0.01865	1.68E-08	12	14	0.03236	4.09E-06	12	14	0.03420	4.09E-06
		s3	7	10	0.02101	8.89E-06	11	13	0.03279	4.14E-06	11	13	0.02879	4.14E-06
		s4	6	8	0.01860	1.63E-07	12	14	0.03087	4.95E-06	12	14	0.03394	4.95E-06
		s5	7	9	0.02043	4.32E-08	12	14	0.03253	5.00E-06	12	14	0.03544	5.00E-06
		s6	5	7	0.01510	1.28E-06	12	14	0.03249	3.10E-06	12	14	0.03180	3.10E-06
	100,000	s1	6	8	0.09285	1.50E-06	13	15	0.19279	3.34E-06	13	15	0.18937	3.34E-06
		s2	6	8	0.09394	5.30E-08	13	15	0.19002	2.58E-06	13	15	0.18880	2.58E-06
		s3	8	11	0.13000	2.83E-08	12	14	0.17314	2.62E-06	12	14	0.17265	2.62E-06
		s4	6	8	0.09615	5.15E-07	13	15	0.19096	3.13E-06	13	15	0.18880	3.13E-06
		s5	7	9	0.11012	1.37E-07	13	15	0.19079	3.16E-06	13	15	0.19892	3.16E-06
		s6	5	7	0.07899	3.98E-06	12	14	0.17753	9.77E-06	12	14	0.17628	9.77E-06

**Table 3:** Numerical experiments for problems 3 & 4 with respect to HAPI, ALG1 & ALG2 methods.

Problem	Dim	IP	HAPI				ALG1				ALG2			
			Iter	Fun	Time(s)	$\left\|𝔉(s_{k})\right\|$	Iter	Fun	Time(s)	$\left\|𝔉(s_{k})\right\|$	Iter	Fun	Time(s)	$\left\|𝔉(s_{k})\right\|$
P3	1,000	s1	30	43	0.01061	8.93E-06	39	47	0.01157	6.28E-06	33	50	0.01060	8.67E-06
		s2	31	44	0.01130	6.63E-06	29	39	0.01000	9.31E-06	33	60	0.01012	8.65E-06
		s3	34	48	0.00892	8.39E-06	31	43	0.01012	8.52E-06	35	60	0.01020	8.65E-06
		s4	29	40	0.00946	9.54E-06	33	43	0.01312	7.63E-06	34	59	0.00986	9.94E-06
		s5	25	33	0.00878	7.47E-06	32	45	0.01394	8.79E-06	35	61	0.01158	7.58E-06
		s6	21	31	0.00648	8.01E-06	22	27	0.00830	7.49E-06	22	34	0.00727	8.49E-06
	10,000	s1	23	31	0.06291	7.90E-06	34	47	0.09532	9.06E-06	35	65	0.10681	8.65E-06
		s2	29	41	0.08868	2.78E-07	36	46	0.09978	6.98E-06	30	50	0.08657	9.26E-06
		s3	25	32	0.06352	9.11E-06	41	56	0.09951	5.41E-06	36	59	0.09862	4.64E-06
		s4	28	39	0.06617	5.18E-06	42	52	0.09271	6.04E-06	35	59	0.10373	8.43E-06
		s5	35	50	0.09721	8.73E-06	38	52	0.10435	6.42E-06	39	63	0.09147	7.67E-06
		s6	14	19	0.03804	6.77E-06	16	20	0.04516	6.35E-06	16	22	0.04607	6.00E-06
	100,000	s1	32	44	0.55067	6.26E-06	34	43	0.60046	9.02E-06	36	63	0.69112	5.75E-06
		s2	21	28	0.36475	7.79E-06	28	38	0.48066	8.23E-06	35	65	0.70745	8.79E-06
		s3	24	33	0.44289	8.67E-06	37	50	0.65476	2.42E-06	42	76	0.81897	7.78E-06
		s4	33	44	0.57989	3.46E-06	43	51	0.72345	2.59E-06	32	53	0.60460	8.01E-06
		s5	32	42	0.54801	8.44E-06	41	54	0.73868	8.56E-06	38	57	0.69986	9.87E-06
		s6	13	19	0.23781	7.94E-06	16	20	0.27747	8.52E-06	17	23	0.31801	1.04E-06
P4	1,000	s1	8	9	0.01647	4.88E-06	10	12	0.00696	3.69E-06	10	12	0.00700	3.69E-06
		s2	6	7	0.00435	3.63E-06	11	14	0.00753	8.06E-06	11	14	0.00736	8.06E-06
		s3	10	12	0.00856	7.62E-06	9	11	0.00707	4.87E-06	9	11	0.00746	4.87E-06
		s4	8	9	0.00634	6.15E-07	8	10	0.00403	9.37E-06	8	10	0.00528	9.37E-06
		s5	9	11	0.00718	9.23E-06	12	15	0.00650	8.08E-06	12	15	0.00758	8.09E-06
		s6	13	14	0.00997	4.24E-07	10	12	0.00716	2.89E-06	11	14	0.00593	7.31E-06
	10,000	s1	5	7	0.02527	9.78E-06	11	13	0.03759	1.49E-06	11	13	0.03979	1.49E-06
		s2	9	11	0.03869	6.12E-07	9	11	0.03362	1.57E-06	9	11	0.03513	1.57E-06
		s3	6	7	0.02506	1.69E-06	10	12	0.03769	9.20E-06	10	12	0.03620	9.20E-06
		s4	6	8	0.02678	6.12E-06	11	13	0.03799	2.78E-06	11	13	0.04142	2.78E-06
		s5	7	8	0.02880	1.88E-06	10	12	0.03504	1.78E-06	10	12	0.03486	1.79E-06
		s6	10	11	0.03770	1.27E-06	9	11	0.03548	5.66E-06	9	11	0.03323	5.63E-06
	100,000	s1	7	9	0.16380	6.11E-06	12	16	0.28022	5.40E-06	12	16	0.26925	5.40E-06
		s2	5	6	0.14323	9.18E-06	9	10	0.19433	7.98E-06	9	10	0.20751	7.98E-06
		s3	10	12	0.21491	2.93E-06	12	14	0.27593	7.79E-06	12	14	0.25239	7.79E-06
		s4	7	9	0.16000	2.96E-06	12	14	0.25411	9.08E-06	12	14	0.25205	9.08E-06
		s5	9	11	0.19724	2.99E-06	10	11	0.23007	7.98E-06	10	11	0.20852	7.98E-06
		s6	6	7	0.12681	6.71E-06	10	11	0.20776	4.26E-07	10	11	0.20937	2.43E-07

**Table 4:** Numerical experiments for problems 5 & 6 with respect to HAPI, ALG1 & ALG2 methods.

Problem	Dim	IP	HAPI				ALG1				ALG2			
			Iter	Fun	Time(s)	$\left\|𝔉(s_{k})\right\|$	Iter	Fun	Time(s)	$\left\|𝔉(s_{k})\right\|$	Iter	Fun	Time(s)	$\left\|𝔉(s_{k})\right\|$
P5	1,000	s1	3	4	0.00561	7.71E-08	10	11	0.00949	7.19E-06	10	11	0.00839	7.19E-06
		s2	3	4	0.00314	8.75E-08	10	11	0.00997	8.16E-06	10	11	0.00841	8.16E-06
		s3	3	4	0.00335	4.23E-08	10	11	0.00960	3.95E-06	10	11	0.00775	3.95E-06
		s4	3	4	0.00222	8.06E-08	10	11	0.00977	7.51E-06	10	11	0.00758	7.51E-06
		s5	3	4	0.00560	6.00E-08	10	11	0.00819	5.59E-06	10	11	0.00768	5.59E-06
		s6	3	4	0.00433	9.42E-08	10	11	0.00683	8.78E-06	10	11	0.00861	8.78E-06
	10,000	s1	3	4	0.01530	2.29E-07	11	12	0.03825	4.54E-06	11	12	0.03662	4.54E-06
		s2	3	4	0.01278	2.60E-07	11	12	0.03490	5.16E-06	11	12	0.03672	5.16E-06
		s3	3	4	0.01699	1.26E-07	11	12	0.03700	2.50E-06	11	12	0.03673	2.50E-06
		s4	3	4	0.01486	2.39E-07	11	12	0.03480	4.75E-06	11	12	0.03722	4.75E-06
		s5	3	4	0.01504	1.77E-07	11	12	0.03507	3.52E-06	11	12	0.03672	3.52E-06
		s6	3	4	0.01366	2.80E-07	11	12	0.03394	5.57E-06	11	12	0.03632	5.57E-06
	100,000	s1	3	4	0.07335	7.23E-07	12	13	0.26499	2.87E-06	12	13	0.27072	2.87E-06
		s2	3	4	0.07272	8.20E-07	12	13	0.24936	3.26E-06	12	13	0.25239	3.26E-06
		s3	3	4	0.07329	3.97E-07	11	12	0.24328	7.89E-06	11	12	0.22784	7.89E-06
		s4	3	4	0.07344	7.55E-07	12	13	0.25494	3.00E-06	12	13	0.24914	3.00E-06
		s5	3	4	0.07576	5.60E-07	12	13	0.26947	2.23E-06	12	13	0.25433	2.23E-06
		s6	3	4	0.07302	8.86E-07	12	13	0.25285	3.52E-06	12	13	0.24700	3.52E-06
P6	1,000	s1	6	8	0.00337	9.30E-07	8	10	0.00812	3.41E-06	8	10	0.00594	3.41E-06
		s2	7	9	0.00712	5.23E-08	12	16	0.00954	3.69E-06	12	16	0.00894	3.69E-06
		s3	8	13	0.00892	4.40E-06	11	14	0.00779	2.71E-06	11	14	0.00695	2.71E-06
		s4	6	8	0.00262	2.65E-08	10	12	0.00705	5.90E-06	10	12	0.00622	5.90E-06
		s5	9	12	0.00848	3.50E-07	11	13	0.00679	4.48E-06	11	12	0.00982	4.45E-06
		s6	5	7	0.00355	6.23E-06	–	–	–	–	–	–	–	–
	10,000	s1	6	8	0.01823	2.94E-06	9	11	0.01881	2.16E-06	9	11	0.01526	2.16E-06
		s2	7	9	0.02536	1.65E-07	13	17	0.02985	2.33E-06	13	17	0.03550	2.33E-06
		s3	9	14	0.02510	1.33E-08	11	14	0.02367	8.56E-06	11	14	0.03343	8.56E-06
		s4	6	8	0.02515	8.37E-08	11	13	0.02637	3.73E-06	11	13	0.02356	3.73E-06
		s5	9	12	0.02837	2.92E-07	11	13	0.02778	4.49E-06	11	12	0.03252	4.44E-06
		s6	6	8	0.02261	2.35E-08	–	–	–	–	–	–	–	–
	100,000	s1	6	8	0.09487	9.30E-06	9	11	0.12070	6.82E-06	9	11	0.12219	6.82E-06
		s2	7	9	0.11590	5.23E-07	13	17	0.19194	7.38E-06	13	17	0.19199	7.38E-06
		s3	9	14	0.16471	4.21E-08	12	15	0.17371	5.41E-06	12	15	0.17622	5.41E-06
		s4	6	8	0.09364	2.65E-07	12	14	0.17606	2.36E-06	12	14	0.17161	2.36E-06
		s5	9	12	0.13100	2.87E-07	11	13	0.14668	4.49E-06	11	12	0.15553	4.44E-06
		s6	6	8	0.10193	7.47E-08	–	–	–	–	–	–	–	–

**Table 5:** Numerical experiments for problems 7 & 8 with respect to HAPI, ALG1 & ALG2 methods.

Problem	Dim	IP	HAPI				ALG1				ALG2			
			Iter	Fun	Time(s)	$\left\|𝔉(s_{k})\right\|$	Iter	Fun	Time(s)	$\left\|𝔉(s_{k})\right\|$	Iter	Fun	Time(s)	$\left\|𝔉(s_{k})\right\|$
P7	1,000	s1	6	7	0.00379	8.76E-08	11	13	0.00229	4.44E-06	11	13	0.00322	4.44E-06
		s2	6	7	0.00175	9.08E-08	10	11	0.00343	2.19E-06	10	11	0.00278	2.19E-06
		s3	7	8	0.00366	1.27E-06	11	13	0.00417	3.05E-06	11	13	0.00430	3.05E-06
		s4	6	7	0.00310	1.36E-07	10	11	0.00488	9.66E-06	10	11	0.00370	9.66E-06
		s5	5	6	0.00377	2.41E-06	9	10	0.00397	5.26E-06	9	10	0.00440	6.67E-06
		s6	6	7	0.00350	6.29E-08	11	12	0.00385	5.34E-06	11	12	0.00411	5.37E-06
	10,000	s1	6	7	0.00968	2.77E-07	12	14	0.01765	2.81E-06	12	14	0.01872	2.81E-06
		s2	6	7	0.00766	2.87E-07	10	11	0.01726	6.94E-06	10	11	0.01582	6.94E-06
		s3	7	8	0.01169	4.01E-06	11	13	0.01632	9.63E-06	11	13	0.01897	9.63E-06
		s4	6	7	0.00918	4.31E-07	11	12	0.01750	6.11E-06	11	12	0.01650	6.11E-06
		s5	5	6	0.00960	2.39E-06	9	10	0.01327	5.27E-06	9	10	0.01507	6.69E-06
		s6	6	7	0.01052	6.16E-08	12	13	0.01801	3.32E-06	12	13	0.01840	3.32E-06
	100,000	s1	6	7	0.05134	8.76E-07	12	14	0.09523	8.87E-06	12	14	0.10341	8.87E-06
		s2	6	7	0.05315	9.08E-07	11	12	0.08979	4.39E-06	11	12	0.08793	4.39E-06
		s3	8	9	0.06502	1.27E-08	12	14	0.09687	6.09E-06	12	14	0.10089	6.09E-06
		s4	6	7	0.04941	1.36E-06	12	13	0.09535	3.86E-06	12	13	0.10182	3.86E-06
		s5	5	6	0.04045	2.39E-06	9	10	0.07245	5.28E-06	9	10	0.07684	6.69E-06
		s6	6	7	0.04986	1.09E-08	13	14	0.09842	2.10E-06	13	14	0.10758	2.1E-06
P8	1,000	s1	6	8	0.00510	5.17E-06	10	13	0.00808	7.17E-06	10	13	0.00730	7.17E-06
		s2	5	7	0.00427	1.76E-06	11	14	0.00869	3.97E-06	11	14	0.00739	3.97E-06
		s3	9	12	0.00577	2.88E-06	14	18	0.00905	6.49E-06	14	18	0.00946	6.49E-06
		s4	6	8	0.00382	4.82E-07	11	14	0.00824	4.61E-06	11	14	0.00648	4.61E-06
		s5	8	10	0.00577	2.34E-07	13	16	0.00896	5.84E-06	13	16	0.00839	5.84E-06
		s6	7	9	0.00491	1.93E-07	11	14	0.00700	3.84E-06	11	14	0.00802	4.05E-06
	10,000	s1	7	9	0.02314	1.66E-08	11	14	0.03142	4.54E-06	11	14	0.03414	4.54E-06
		s2	5	7	0.01707	5.57E-06	12	15	0.03208	2.51E-06	12	15	0.03584	2.51E-06
		s3	9	12	0.02832	9.11E-06	15	19	0.04622	4.10E-06	15	19	0.04748	4.1E-06
		s4	6	8	0.01899	1.52E-06	12	15	0.03614	2.92E-06	12	15	0.03874	2.92E-06
		s5	8	10	0.02486	8.20E-07	14	17	0.04489	3.77E-06	14	17	0.03961	3.77E-06
		s6	7	9	0.02296	1.91E-07	11	14	0.03146	3.84E-06	11	14	0.03425	4.05E-06
	100,000	s1	7	9	0.12550	5.24E-08	12	15	0.21062	2.87E-06	12	15	0.21762	2.87E-06
		s2	6	8	0.10540	1.79E-08	12	15	0.20447	7.94E-06	12	15	0.20239	7.94E-06
		s3	10	13	0.18698	2.93E-08	16	20	0.27920	2.60E-06	16	20	0.28477	2.6E-06
		s4	6	8	0.11383	4.82E-06	12	15	0.21068	9.22E-06	12	15	0.20518	9.22E-06
		s5	8	10	0.14323	2.63E-06	15	18	0.25251	2.39E-06	15	18	0.25640	2.39E-06
		s6	7	9	0.11567	1.91E-07	11	14	0.19098	3.84E-06	11	14	0.17933	4.04E-06

**Table 6:** Numerical experiments on the HAPI and HAPM algorithms for problems 1 to 8.

Problem	n−value	IP	HAPI				HAPM			
			Iter	Fun	Time(s)	$\left\|𝔉(s_{k})\right\|$	Iter	Fun	Time(s)	$\left\|𝔉(s_{k})\right\|$
1	100,000	s1	7	11	0.00412	5.71E-07	9	13	0.17227	3.71E-06
		s2	6	8	0.00424	2.18E-07	12	14	0.15800	2.50E-06
		s3	9	13	0.00546	1.69E-07	11	15	0.15586	1.85E-06
		s4	9	11	0.00526	9.77E-07	8	12	0.13933	6.36E-06
		s5	9	13	0.00514	4.64E-06	14	20	0.20505	6.48E-06
		s6	12	16	0.00476	1.36E-06	17	22	0.24958	9.58E-06
2		s1	6	8	0.00513	3.81E-06	10	12	0.15015	2.91E-06
		s2	5	7	0.00403	9.66E-07	10	12	0.15098	1.14E-06
		s3	8	11	0.00665	3.75E-07	11	14	0.17526	5.17E-06
		s4	6	8	0.00420	1.38E-06	10	12	0.15781	2.21E-06
		s5	7	9	0.00498	1.28E-06	10	12	0.15433	5.16E-06
		s6	5	7	0.00389	8.28E-06	9	11	0.14374	5.04E-06
3		s1	32	44	0.01176	8.61E-07	34	50	0.60266	9.48E-06
		s2	35	50	0.01495	6.06E-06	29	42	0.51188	3.09E-06
		s3	26	36	0.00856	7.41E-06	28	43	0.51546	6.76E-06
		s4	22	30	0.01194	5.20E-06	29	40	0.51042	6.10E-06
		s5	20	26	0.00832	9.78E-06	34	48	0.58294	2.29E-06
		s6	17	24	0.00521	6.76E-06	15	21	0.26370	6.36E-06
4		s1	6	8	0.00613	8.44E-07	8	10	0.17445	8.00E-07
		s2	8	10	0.00639	2.76E-07	10	12	0.27396	1.80E-06
		s3	6	8	0.00490	2.31E-06	10	12	0.22117	3.66E-06
		s4	6	8	0.00590	7.04E-07	10	12	0.21931	5.71E-06
		s5	5	6	0.00527	7.41E-07	7	8	0.15482	9.78E-06
		s6	9	10	0.00524	7.71E-08	6	8	0.14339	8.37E-06
5		s1	4	5	0.00281	7.09E-07	8	9	0.17189	7.01E-06
		s2	4	5	0.00510	8.05E-07	8	9	0.18267	7.96E-06
		s3	4	5	0.00521	3.89E-07	8	9	0.17387	3.85E-06
		s4	4	5	0.00593	7.41E-07	8	9	0.18095	7.33E-06
		s5	4	5	0.00461	5.52E-07	8	9	0.22738	5.43E-06
		s6	4	5	0.00400	8.67E-07	8	9	0.17468	8.60E-06
6		s1	6	8	0.00735	1.36E-06	9	11	0.13101	4.02E-06
		s2	7	9	0.00574	2.18E-06	10	13	0.17044	5.39E-06
		s3	9	14	0.00692	1.75E-07	10	13	0.17684	3.24E-06
		s4	5	7	0.00546	8.45E-06	8	10	0.12048	4.77E-06
		s5	9	12	0.00883	1.61E-06	9	11	0.15152	6.65E-06
		s6	5	7	0.00622	9.13E-06	9	11	0.14983	3.36E-06
7		s1	6	7	0.00304	3.72E-06	9	10	0.07927	9.54E-06
		s2	6	7	0.00239	4.06E-06	9	10	0.07706	8.76E-06
		s3	8	9	0.00297	1.79E-07	11	13	0.09649	2.61E-06
		s4	6	7	0.00307	5.54E-06	10	11	0.08445	1.29E-06
		s5	6	7	0.00334	1.15E-07	7	8	0.06660	2.39E-06
		s6	6	7	0.00174	2.14E-06	9	10	0.07690	4.99E-06
8		s1	6	8	0.00664	4.94E-07	9	12	0.16337	1.39E-06
		s2	6	8	0.00489	7.03E-07	8	11	0.14311	6.47E-06
		s3	10	13	0.00681	1.82E-06	14	17	0.26761	2.31E-06
		s4	6	8	0.00579	9.07E-06	8	11	0.15119	9.74E-06
		s5	9	11	0.00480	1.29E-07	12	15	0.22030	5.59E-06
		s6	7	9	0.00550	2.71E-06	9	11	0.14993	3.07E-06

**Fig 1:**
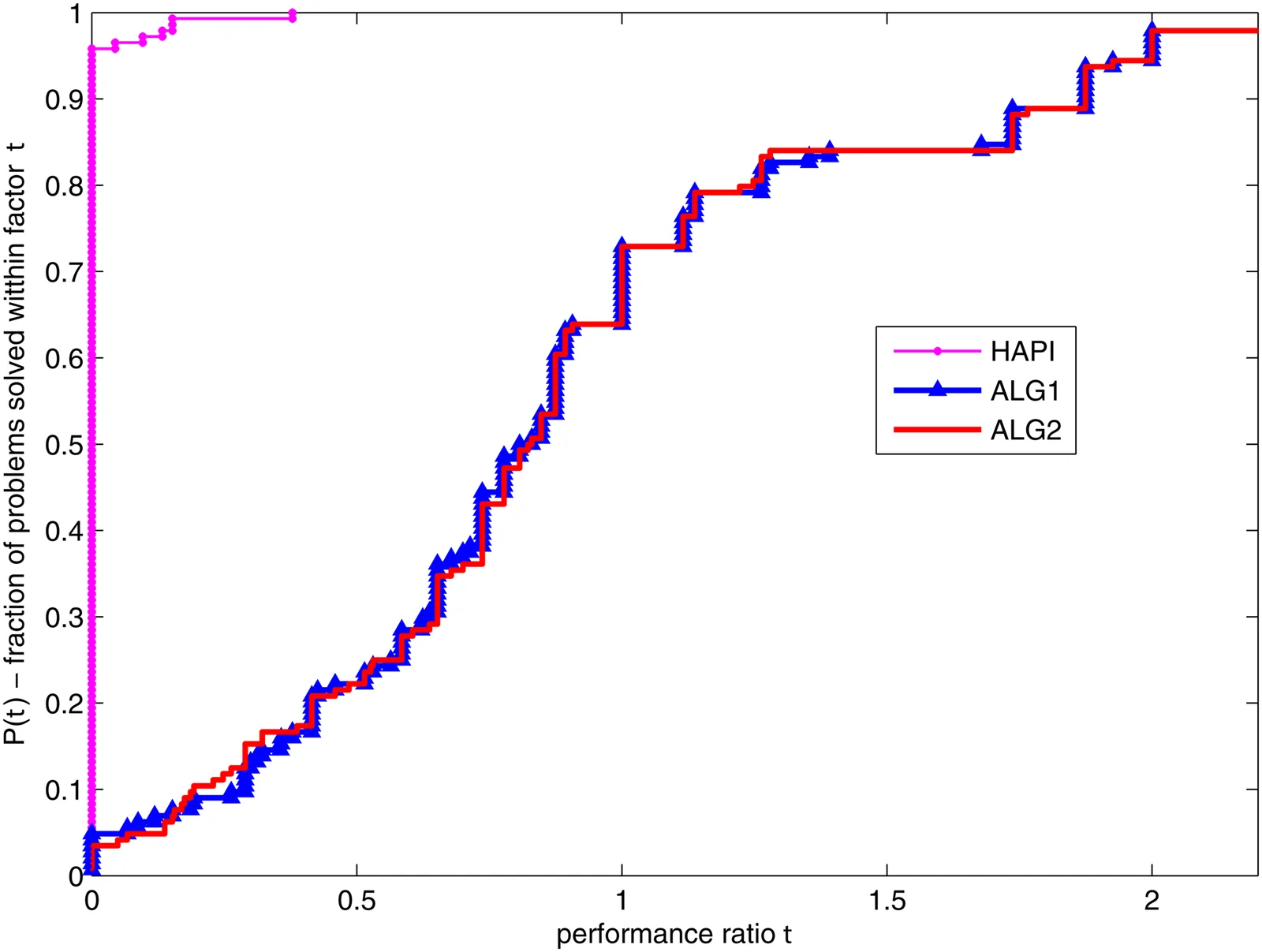
Performance profile based on the number of iterations within a factor τ of the best time.

**Fig 2:**
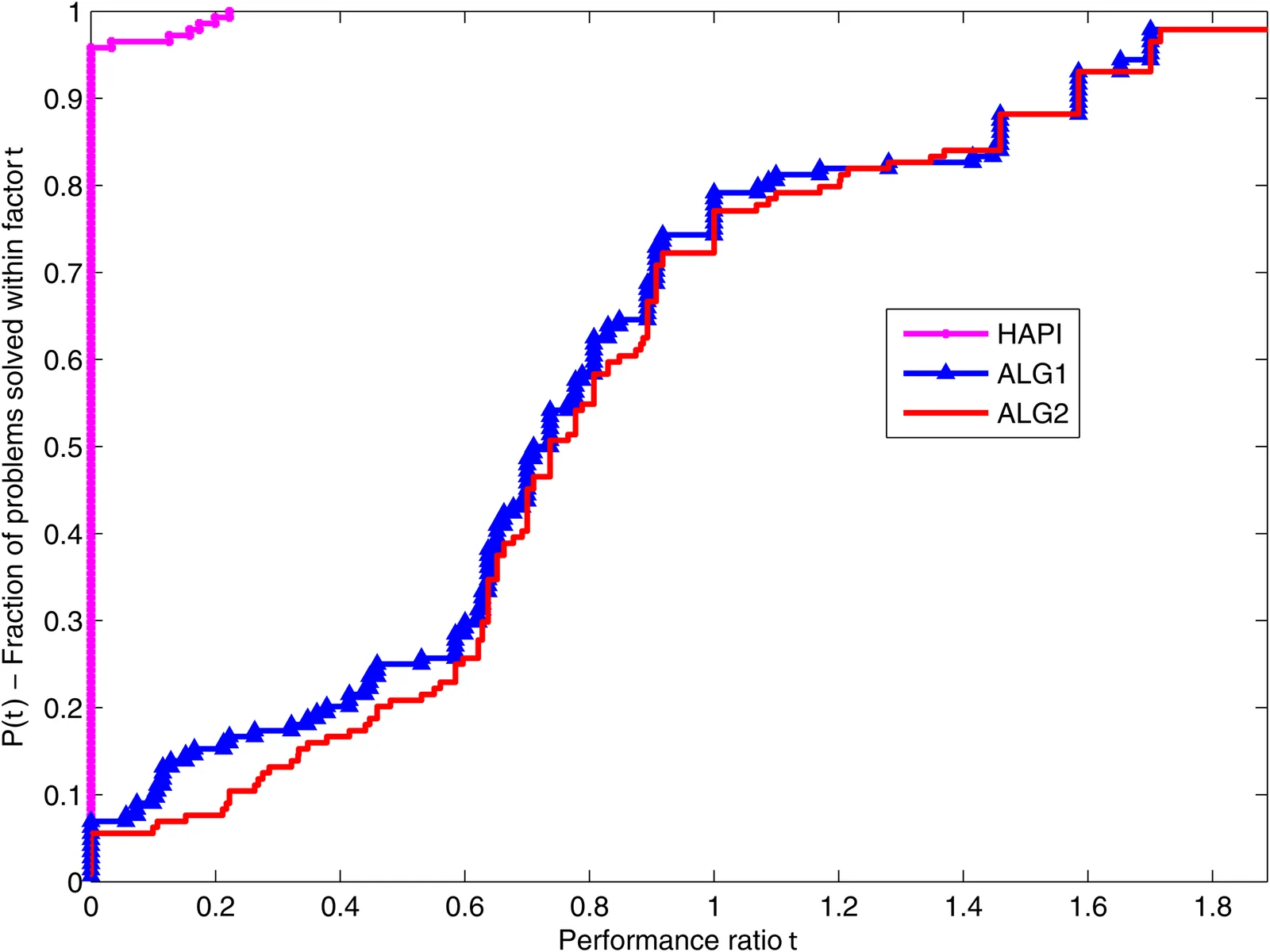
Performance profile based on the function evaluation within a factor τ of the best time.

**Fig 3:**
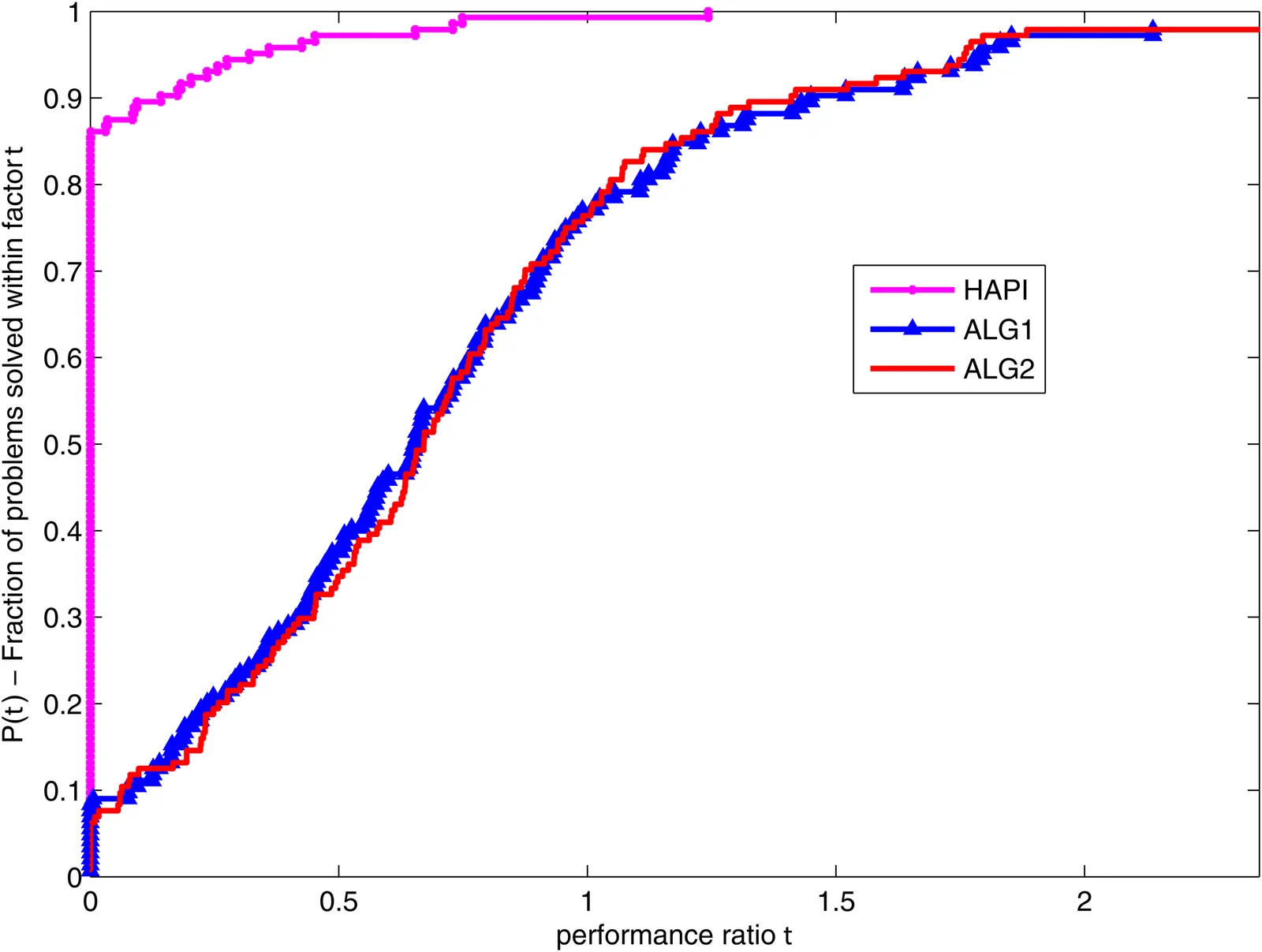
Performance profile with respect to CPU time (in seconds) within a factor τ of the best time.

Figs 1–3 present the performance of the three methods using the profile of Dolan and Mor$\overset{´}{e}$ [45], with respect to the number of iterations required to solve the test problems. The horizontal axis displays the performance ratio τ, and the vertical axis gives the fraction P(τ) of problems for which each method converges within a factor τ of the best iteration count, function evaluation, and CPU time (in seconds). The figures show that HAPI consistently outperforms the other two methods. As τ increases, the curve for the proposed method stays consistently above the curves for the ALG1 and ALG2 approaches. This means that the HAPI method successfully solves the largest fraction of problems using the fewest iterations, function evaluations, and CPU time, demonstrating its superior robustness across the test set. In particular, HAPI converges more quickly to the solution than the other methods, ensuring its overall dependability and competitiveness. The results indeed indicate that incorporating the hybridisation process and the Jacobian approximation with an acceleration parameter yields a method that not only converges faster but is also more stable compared to the existing approaches.

Table 6 presents the numerical results of the HAPI and HAPM algorithms for Problems 1–8 using different initial points and a fixed dimension *n* = 100,000. The comparison is carried out in terms of the number of iterations, function evaluations, computational time, and residual norms. The results show that both methods converged successfully for all test problems with satisfactory accuracy. However, the HAPI algorithm generally required fewer iterations, fewer function evaluations, and less CPU time than HAPM. This demonstrates the efficiency of the acceleration strategy incorporated in HAPI. Furthermore, the residual norms obtained by HAPI are comparable to those of HAPM, indicating that the improved computational performance is achieved without loss of accuracy. Overall, the results confirm that HAPI is more efficient and robust than HAPM for the considered test problems.

**Fig 4:**
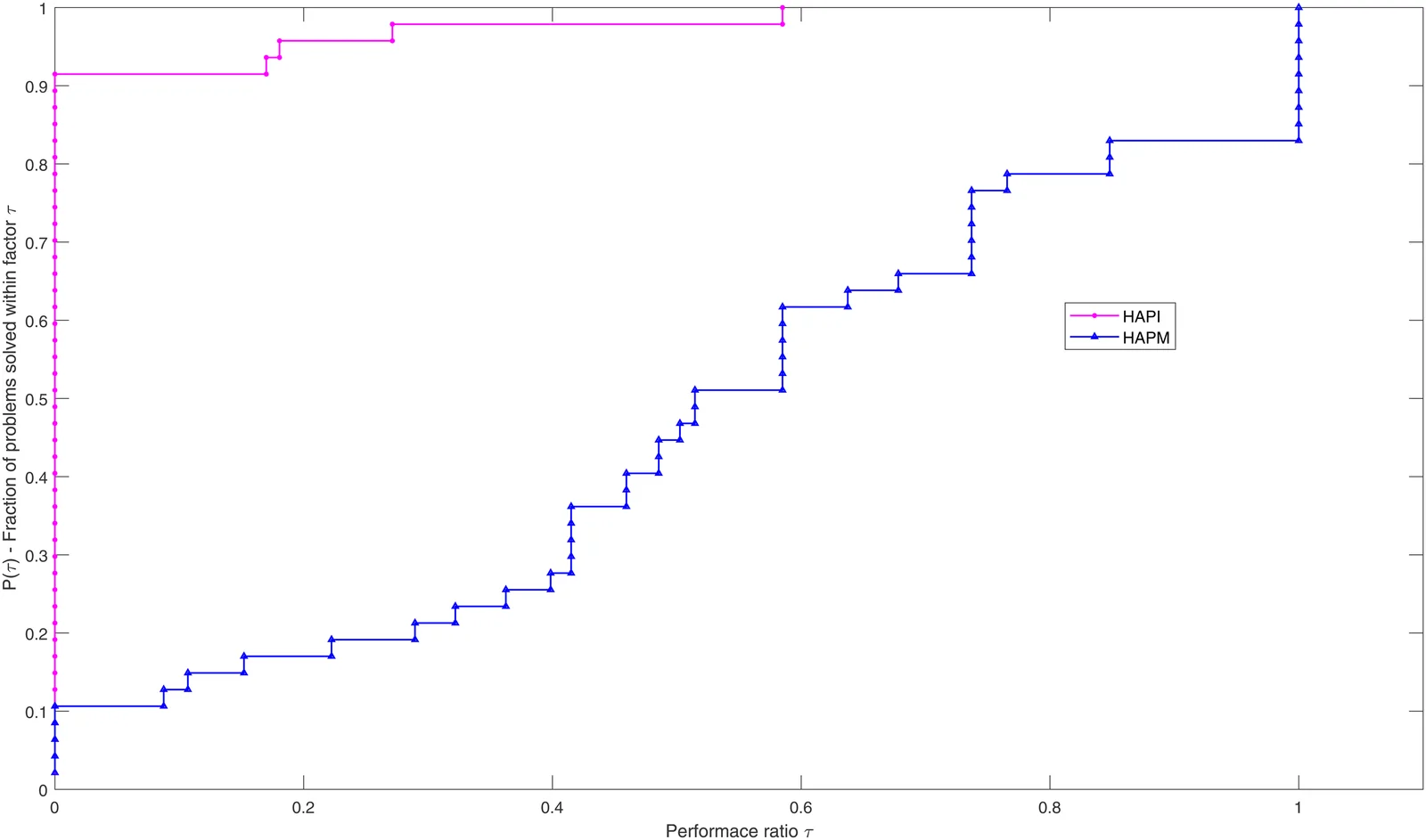
Performance profile based on the number of iterations within a factor τ of the best time.

**Fig 5:**
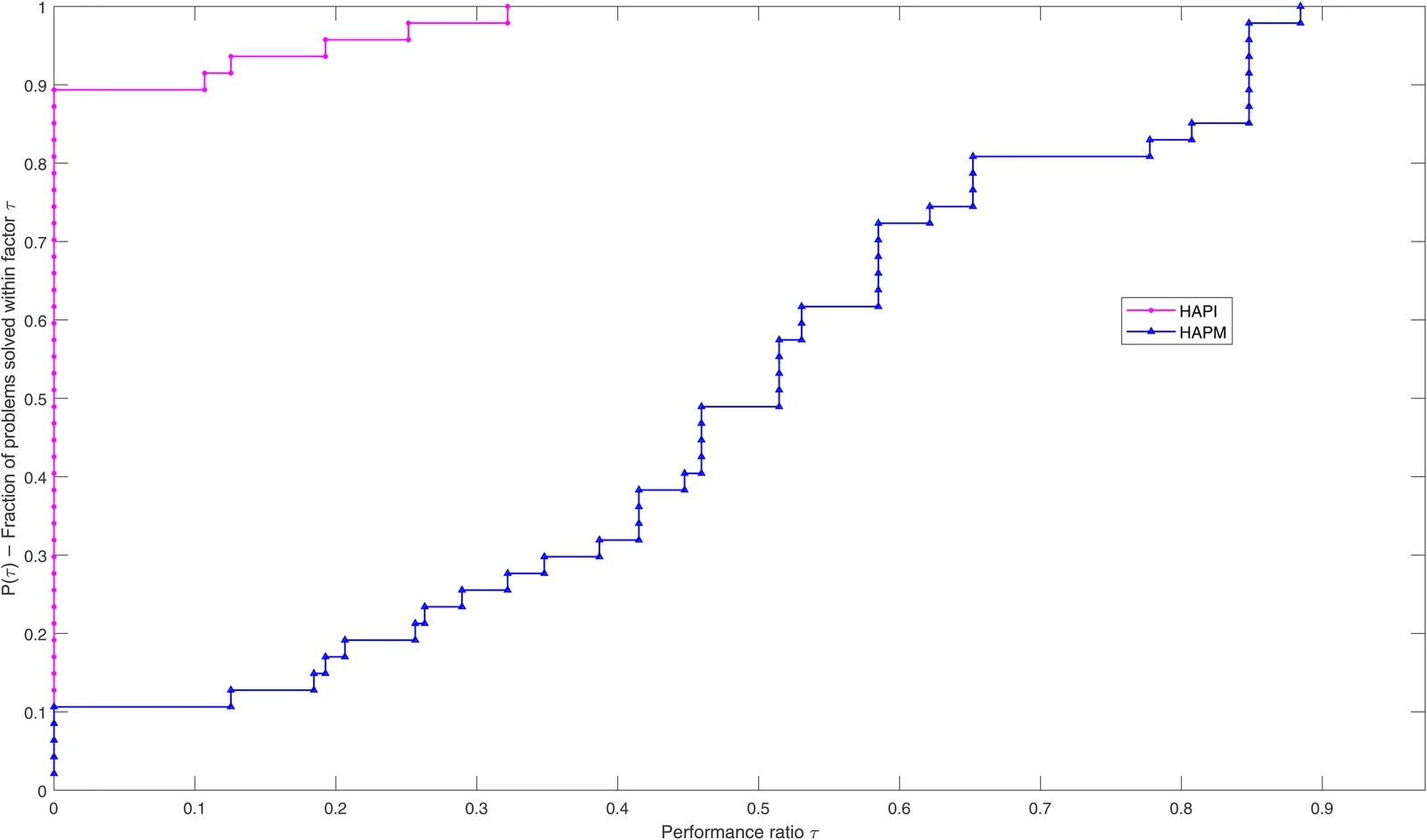
Performance profile based on the function evaluation within a factor τ of the best time.

Fig 4 and Fig 5 present the performance of the three methods using the profile of Dolan and Mor$\overset{´}{e}$ [45], with respect to the number of iterations, function evaluations, and CPU time. The performance profile shows that HAPI performs better than HAPM on most test problems. The HAPI curve rises rapidly indicating that it solves almost all problems with the best or near-best performance. In contrast, HAPM requires larger performance ratios before solving the same fraction of problems, showing lower efficiency and robustness. Since the HAPI curve lies above and to the left of the HAPM curve, HAPI is considered the superior algorithm according to the Dolan and Mor$\overset{´}{e}$ criterion.

## 5. Conclusion

**Fig 6:**
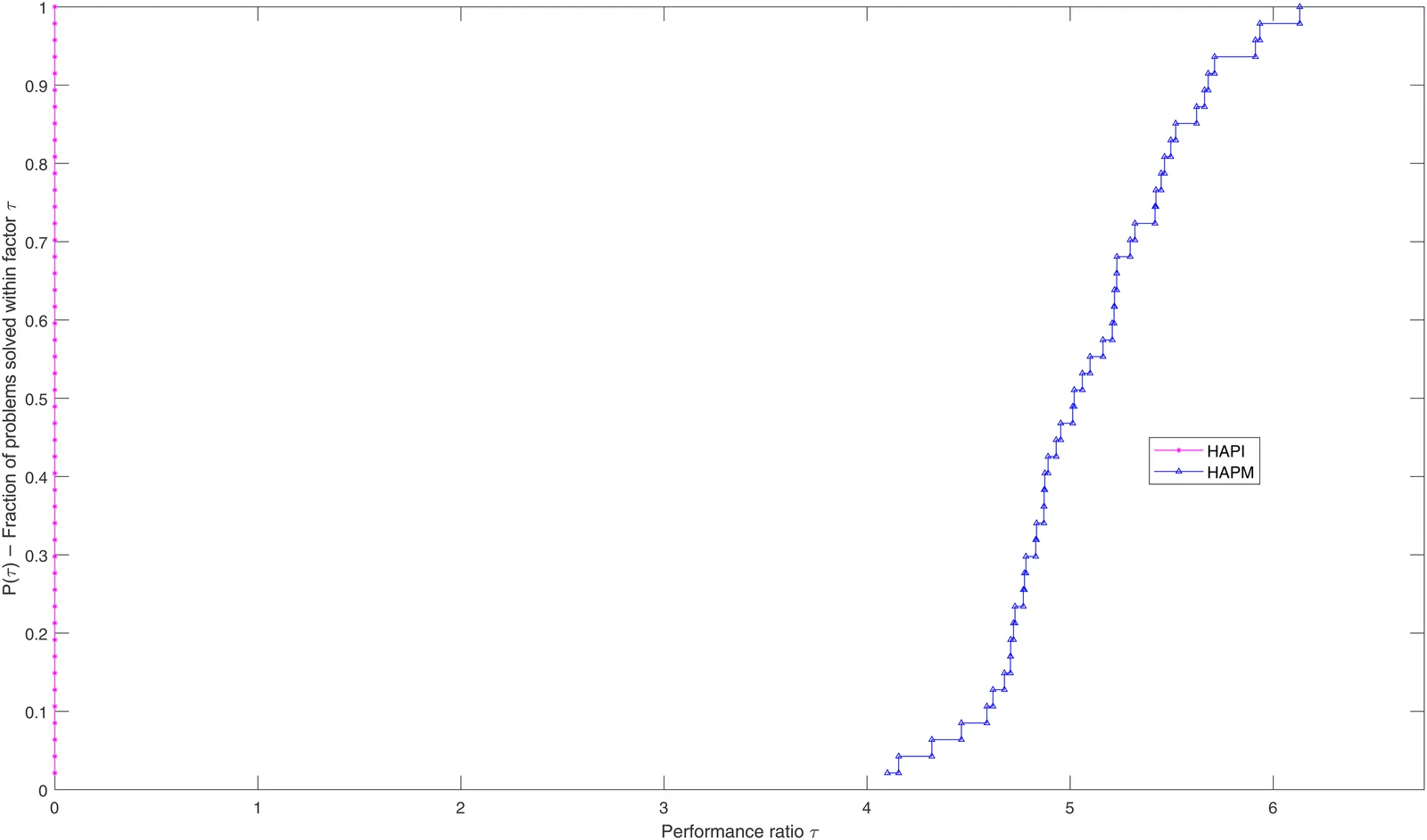
Performance profile with respect to CPU time (in seconds) within a factor τ of the best time.

This paper presents an accelerated approach for solving systems of nonlinear equations. The proposed method combines the Picard–Ishikawa process with an acceleration scheme and introduces a correction parameter to reduce computational cost while maintaining high accuracy. Furthermore, the acceleration parameter was derived to approximate the Jacobian matrix effectively. Under mild assumptions, the theoretical analysis established the global convergence of the proposed method. Extensive numerical experiments were carried out on several benchmark problems to evaluate the efficiency and robustness of the proposed scheme. The numerical results presented in Tables 2–6 and Fig 1–Fig 6 demonstrate that the proposed HAPI method consistently outperforms the existing ALG1, ALG2, and HAPM methods in terms of iteration count, function evaluations, and CPU time while preserving high numerical accuracy. In particular, the Dolan and Mor$\overset{´}{e}$ performance profiles confirm the superiority and robustness of the HAPI method, as it solves most test problems with the best or near-best performance.

The main contribution of this work lies in the development of a robust hybrid accelerated iterative framework based on the Picard–Ishikawa process for efficiently solving large-scale nonlinear systems. The proposed approach improves convergence behavior and computational efficiency while avoiding direct Jacobian evaluations.

Although the proposed method has demonstrated promising numerical performance on a variety of benchmark problems, the current study is limited to systems of nonlinear equations that satisfy the assumptions required for the convergence analysis. Therefore, its performance on more challenging classes of problems, such as highly ill-conditioned or large-scale real-world applications, requires further investigation. Overall, the obtained results validate the effectiveness and applicability of the proposed accelerated scheme for solving nonlinear systems of equations arising in scientific and engineering applications. Future research will focus on exploring alternative acceleration strategies and hybrid iterative processes to further improve convergence behavior and computational efficiency. In addition, future studies will investigate the applicability of the proposed method to practical problems arising in motion control and related engineering design, and other real-world optimization applications will be investigated.
